# Dickkopf 1 is expressed in normal fibroblasts during early stages of colorectal tumorigenesis

**DOI:** 10.1002/cam4.6992

**Published:** 2024-02-09

**Authors:** Yushi Inomata, Masatake Kuroha, Yusuke Shimoyama, Takeo Naito, Rintaro Moroi, Hisashi Shiga, Yoichi Kakuta, Hideaki Karasawa, Shinobu Onuma, Yoshitaka Kinouchi, Atsushi Masamune

**Affiliations:** ^1^ Division of Gastroenterology Tohoku University Graduate School of Medicine Sendai Japan; ^2^ Department of Surgery Tohoku University Graduate School of Medicine Sendai Japan; ^3^ Student Healthcare Center, Institute for Excellence in Higher Education Tohoku University Sendai Japan

**Keywords:** colorectal adenoma, colorectal cancer, Dickkopf 1, fibroblast, organoid

## Abstract

**Background and Purpose:**

Colorectal cancer progression from adenoma to cancer is a time‐intensive process; however, the interaction between normal fibroblasts (NFs) with early colorectal tumors, such as adenomas, remains unclear. Here, we analyzed the response of the microenvironment during early tumorigenesis using co‐cultures of organoids and NFs.

**Materials and Methods:**

Colon normal epithelium, adenoma, cancer organoid, and NFs were established and co‐cultured using Transwell inserts. Microarray analysis of NFs was performed to identify factors expressed early in tumor growth. Immunostaining of clinical specimens was performed to localize the identified factor. Functional analysis was performed using HCT116 cells. Serum DKK1 levels were measured in patients with colorectal cancer and adenoma.

**Results:**

Colorectal organoid–NF co‐culture resulted in increased organoid diameter and cell viability in normal epithelial and adenomatous organoids but not in cancer organoids. Microarray analysis of NFs revealed 18 genes with increased expression when co‐cultured with adenoma and cancer organoids. Immunohistochemical staining revealed *DKK1* expression in the tumor stroma from early tumor growth. *DKK1* stimulation reduced HCT116 cell proliferation, while *DKK1* silencing by siRNA transfection increased cell proliferation. Serum DKK1 level was significantly higher in patients with advanced cancer and adenoma than in controls. Serum DKK1 level revealed area‐under‐the‐curve values of 0.78 and 0.64 for cancer and adenoma, respectively.

**Conclusion:**

These findings contribute valuable insights into the early stages of colorectal tumorigenesis and suggest *DKK1* as a tumor suppressor. Additionally, serum DKK1 levels could serve as a biomarker to identify both cancer and adenoma, offering diagnostic possibilities for early‐stage colon tumors.

The present study has a few limitations. We considered using DKK1 as a candidate gene for gene transfer to organoids and NFs; however, it was difficult due to technical problems and the slow growth rate of NFs. Therefore, we used cancer cell lines instead. In addition, immunostaining and ELISA were based on the short‐term collection at a single institution, and further accumulation of such data is desirable. As described above, most previous reports were related to advanced cancers, but in this study, new findings were obtained by conducting experiments on endoscopically curable early‐stage tumors, such as adenomas.

## Graphical abstract

The concentration of DKK1 was found to be increased by co‐culture with tumor organoids than when NFs were cultured alone. Furthermore, we measured serum DKK1 in patients with colorectal tumors to evaluated its usefulness as biomarker, The analysis revealed significantly higher serum DKK1 levels in the advanced cancer patient group than in the control group; th AUC was 0.78. Moreover, serum DKK1 concentration before and after treatment in patients with advanced adenoma showed a decrease in the after‐treatment group compared with that in the before‐treatment group; the AUC was 0.64.

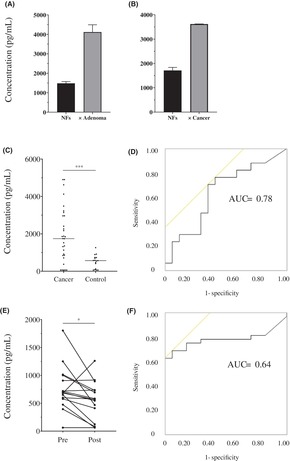

## 1 INTRODUCTION

Colorectal cancer is one of the most common cancers worldwide and the leading cause of cancer‐related deaths.^1^, ^2^, ^3^ The prognosis for patients at the early stage is satisfactory but is poor for patients at advanced stages.^4^ The tumor microenvironment (TME) in colorectal cancer comprises fibroblasts, inflammatory cells, blood vessels, nerves, extracellular matrix (ECM), and basement membrane.^5^ The TME plays a crucial role in colorectal cancer carcinogenesis and acts as a therapeutic target and prognostic factor.^6^ Among TME components, cancer‐associated fibroblasts (CAFs) are key functional regulators that interact with cancer cells.^7^ CAFs, a heterogeneous cell population,^5^ exhibit diverse functions and have been implicated in reduced responsiveness to immunotherapy.^8^, ^9^ Transforming growth factor‐β stimulation,^10^, ^11^ inflammatory cytokines, and receptor tyrosine kinase ligands collectively contribute to the formation of CAFs.^12^ Normal fibroblasts (NFs) surrounding the cancer cells are considered the progenitors of CAFs.^13^ However, the precise process of CAF formation from NFs remains unclear. The abundance of NFs in precancerous lesions and early‐stage cancer lesions^14^, ^15^ suggests their potential role during the early stages of tumorigenesis. However, research on this topic is scarce, possibly because of the difficulty of sampling and observing lesions in human tissues over time and the lack of a suitable model for observing the interrelationship between fibroblasts and early lesions or precancerous lesions.

Traditionally, in vitro studies of colorectal cancer relied on cancer cell lines due to the unavailability of technology for culturing normal intestinal epithelial cells or colorectal adenomas.^16^ However, the discovery of the technique for culturing three‐dimensional structures called organoids has contributed to advancements in in vitro models for various studies. These organoids obtained by isolation of crypts from mouse small intestines have been proven versatile for use not only in mice but also in humans, extending their application to tumor studies.^17^, ^18^ However, a limitation of the organoid model was the absence of TME components in the culture system.^19^ Recent advancements, such as co‐culture systems,^20^, ^21^, ^22^ have shown promise to address these limitations, leading to the development of a simple model to clarify the interrelationship between tumors and TME components.

We hypothesized that the organoid culture technique and the co‐culture model of fibroblasts could be used to analyze the responses of the microenvironment at the early stage of colorectal tumorigenesis. To test this hypothesis, we aimed to analyze the response of the TME during early tumorigenesis using co‐cultures of organoids and NFs. In addition, we also identified serum markers of colorectal tumors using early response factors.

## 2 RESULTS

### 2.1 Co‐culture of organoids and NFs

**FIGURE 1:**
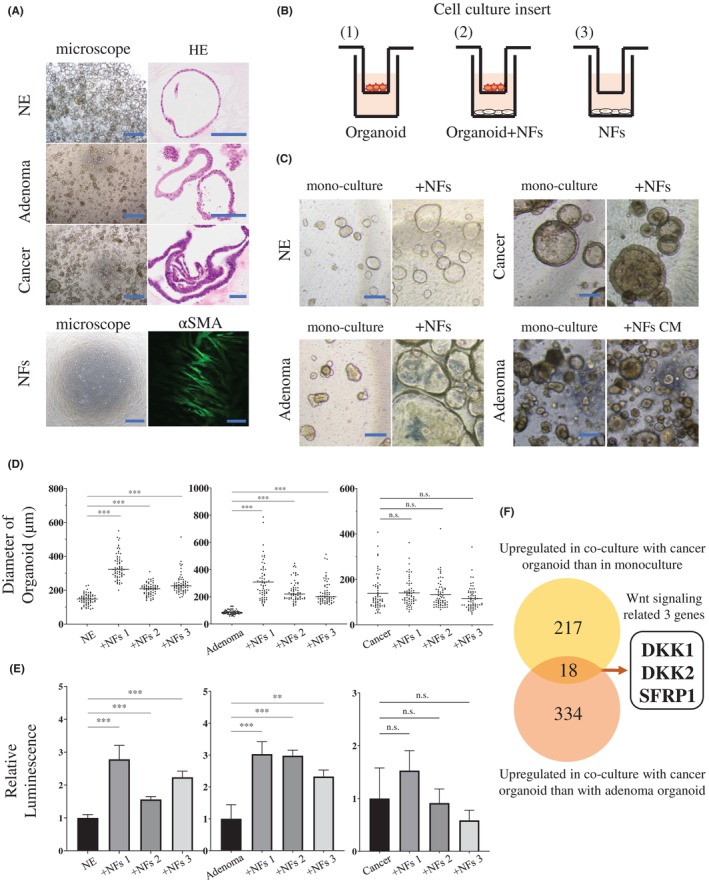
Co‐culture of colon organoids and normal fibroblasts. (A) Colon organoids (derived from NE, adenoma, and cancer tissues). Left row: Optical microscope image. Scale bars, 500 μm. Right row: H&E‐stained, Scale bars, 100 μm. (B) NFs derived from NE. Left: Optical microscope image. Scale bars, 500 μm. Right: fluorescent immunostaining with *αSMA* observed using confocal laser microscopy, Scale bar: 100 μm. (C) Schematic diagram of co‐culture or monoculture using Transwell inserts. (C) Optical microscopic images of co‐cultured NE, cancer, and adenoma organoids (upper left, right, and lower left, respectively) in the insert on Day 10. Lower right, adenoma organoids cultured with NF‐conditioned medium on Day 7. Scale bars: 200 μm. (D) Diameter of organoid in the insert after co‐culture. (E) Cell viability of organoid after co‐culture. (F) Verification of gene expression profiles using microarray analysis of NFs co‐cultured with adenoma or cancer organoids. The numbers of genes upregulated in co‐cultures with cancer organoids compared to those in monocultures (upper circle) and co‐cultures with adenoma organoids (lower circle) are shown. In (D) and (E), **p < 0.01; ***p < 0.001. CM, conditioned medium; NE, normal epithelium; NFs, normal fibroblasts.

Organoids derived from colon normal epithelium, adenomatous, and cancer tissues obtained from patients with colorectal cancer (Table 1) were established (Figure 1A). The intestinal epithelium‐like structures of each established organoid were confirmed using H&E staining (Figure 1A, right). Organoids showed irregular glandular structures and increased nucleus‐to‐cytoplasm ratio with the progression in tumor grades. In this study, NFs were established using three cell lines (Table 1); the NFs stained positive for *αSMA* (Figure 1A, lower panels) but not for the epithelial marker E‐cadherin with fluorescent immunostaining. Organoids and NFs were cultured independently or together, as shown in Figure 1B. The optical microscopic images of the co‐cultured organoids on Day 10 are shown in Figure 1C. The adenoma and normal epithelium organoids co‐cultured with NFs did not show apparent morphological changes among the three NF cell lines; however, the mean diameter of the adenoma organoids co‐cultured with these NFs (308.3, 242.9, and 227.1 μm, as assessed using three specimens each) differed significantly from that of the monoculture adenoma organoids (86.5 μm) (*p* < 0.001; Figure 1D). Similar trends of increased diameter were observed in co‐cultured normal epithelium organoids (338.9, 205.8, and 236.3 μm, respectively; *n* = 3, each) compared with those in monoculture organoids (146.2 μm; *p* < 0.001; Figure 1D). In contrast, no significant changes were observed between cancer organoids mono‐ (138.1 μm) and co‐cultures (140.2 μm, *p* = 0.855; 133.5 μm, *p* = 0.706; 115.9 μm, *p* = 0.052; respectively in three NF cell lines; *n* = 3, each; Figure 1D). For the evaluation of cell viability, the relative values were calculated by setting the mean of the measured values in the monoculture group of each organoid as 1. The mean relative luminescence values after co‐culture with normal epithelium organoids (2.78, 1.56, and 2.24, respectively; *p* < 0.001 in all cases) increased significantly compared to that in their monocultures. Similarly, the adenoma organoids co‐cultures showed increased luminescence values (3.03, *p* < 0.001; 2.98, *p* < 0.001; and 2.33, *p* = 0.002; respectively) compared to that in their monocultures. However, the mean relative luminescence values of cancer organoids after co‐culture with the three NFs did not differ significantly (Figure 1E).

**TABLE 1:** Identified 18 overexpressed genes.

Gene symbol	Gene name
*AAK1*	AP2 associated kinase 1
*ACTG2*	Actin, gamma 2, smooth muscle
*BLID*	BH3‐like motif containing, cell death inducer
*BRCC3*	BRCA1/BRCA2‐containing complex, subunit 3
*CLDN11*	Claudin 11
*CLEC4GP1*	C‐type lectin domain family 4, member G pseudogene 1
*DKK1*	Dickkopf 1
*DKK2*	Dickkopf 2
*DYSF*	Dysferlin, limb girdle muscular dystrophy 2B
*OXTR*	Oxytocin receptor
*PCDH9*	Protocadherin 9
*POU2AF1*	POU class 2 associating factor 1
*RP1L1*	Retinitis pigmentosa 1‐like 1
*SFRP1*	Secreted frizzled related protein 1
*SFTA1P*	Surfactant associated 1, pseudogene
*SNORA74A*	Small nucleolar RNA, H/ACA box 74A
*SNORD67*	Small nucleolar RNA, C/D box 67
*TUT4*	Terminal uridylyl transferase 4

NFs respond differently depending on the tumor stage of the organoid in which they are co‐cultured; therefore, we performed a microarray analysis of the co‐cultured NFs to comprehensively identify the genes with variable expression. A total of 217 genes were identified to be upregulated in NFs co‐cultured with cancer organoids compared to NF monocultures. Additionally, 334 genes were upregulated in NFs co‐cultured with cancer organoids compared to those in NFs co‐cultured with adenoma organoids (Data S1). We hypothesized that co‐culturing NFs would progressively enhance the expression of genes involved in the suppression of tumor development from adenoma to cancer and searched for the common genes. As a result, 18 genes were identified (Figure 1F and Table 2), of which three genes—*DKK1*, *DKK2*, and *SFRP1*—related to Wnt signaling were further investigated (Figure 1F).

**TABLE 2:** Patient background of clinical specimens immunostained for *DKK1.*

	Nonadvanced adenoma (*n* = 27)	Advanced adenoma (*n* = 25)	Advanced cancer (*n* = 56)
Average age (range)	68.9 (41–82)	70.0 (42–87)	68.2 (34–92)
Sex			
Male (%)	17 (63.0)	14 (56)	28 (50.0)
Female (%)	10 (37.0)	11 (44)	28 (50.0)
Location			
Right colon (%)	12 (44)	13 (52)	21 (37.5)
Left colon (%)	15 (56)	12 (48)	35 (62.5)

### 2.2 Immunohistochemical staining using resection specimens

**FIGURE 2:**
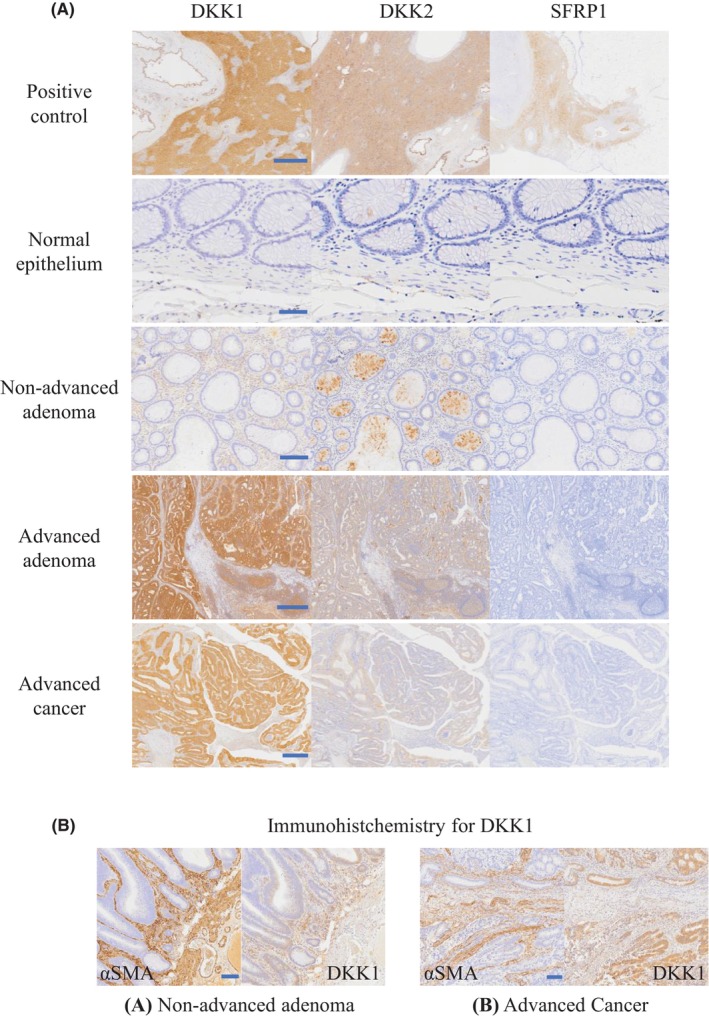
Immunohistochemical staining of *DKK1* in clinical specimens. (A) Representative images of *DKK1* in human liver tissue, *DKK2* in human hepatocarcinoma tissue, *SFRP1* in human heart muscle tissue in positive control (scale bar, 1000 μm), normal epithelium (scale bar, 50 μm), nonadvanced adenoma (scale bar, 200 μm), advanced adenoma (scale bar, 500 μm), and advanced cancer (scale bar, 500 μm). (B) Comparison of specimens with immunohistochemical staining for *αSMA* and *DKK1* in (a) nonadvanced adenoma and (b) advanced cancer. Scale bars: 100 μm. *DKK1*, Dickkopf‐1; *αSMA*, α‐smooth muscle actin.

Expression of *DKK1*, *DKK2*, and *SFRP1* was verified by IHC staining of clinical specimens of colon normal epithelium, adenomatous, and cancer tissues. All three proteins were expressed in the positive control (Figure 2A) but not in the normal epithelium (Figure 2A). The expression of *SFRP1* was not observed in advanced and nonadvanced adenoma or advanced cancer specimens (Figure 2A). For *DKK2*, nonspecific staining in the tumor duct was observed in nonadvanced adenoma; however, staining of the tumor adenoduct and stroma was observed in advanced adenoma (Figure 2A). *DKK1* was expressed predominantly in the tumor stroma in nonadvanced adenomas, with a trend toward predominant expression in the tumor adenoduct beginning in advanced adenomas (Figure 2A). IHC staining of the same specimens for *DKK1* and *αSMA* revealed that in nonadvanced adenoma, *αSMA* stained at the *DKK1*‐staining site in the tumor stroma (Figure 2B‐a), whereas in advanced cancer, the staining sites did not match, and *DKK1* tended to concentrate in the tumor adenoduct (Figure 2B‐b).

As the objective was to evaluate the response in the early stages of tumor development, we evaluated the expression of *DKK1* by IHC staining in 27 nonadvanced adenomas, 25 advanced adenomas, and 56 advanced cancer specimens. The background of the patients from whom these specimens were collected is shown in Table 3. The cutoff values for negative and positive results were defined by the mean expression score of all specimens (4.09 points in the adenoductal area and 2.86 points in the stromal area). As shown in Table 4, the positivity rate increased substantially with the progression of tumor malignancy (nonadvanced adenoma–advanced adenoma: *p* < 0.001, advanced adenoma–advanced cancer: *p* = 0.009) in the adenoductal area. No significant change was observed between nonadvanced adenoma and advanced adenoma in the stromal area; however, a significant decrease in the positivity rate was observed with the tumor progression from advanced adenoma to advanced cancer (nonadvanced adenoma–advanced adenoma: *p* = 0.432, advanced adenoma–advanced cancer: *p* = 0.008).

**TABLE 3:**
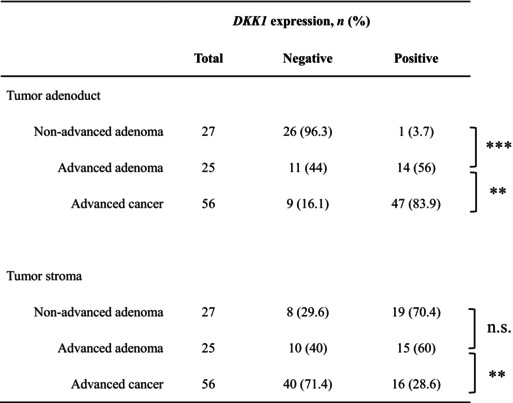
Histological expression of *DKK1* in resection specimens.

Abbreviation: n.s., not significant.

** *p* < 0.01.

*** *p* < 0.001.

**TABLE 4:** Patient background of serum used for ELISA.

	Advanced adenoma (*n* = 17)	Advanced cancer (*n* = 32)
Average age (range)	67.5 (49–81)	64.3 (48–79)
Sex		
Male (%)	7 (41.2)	15 (46.9)
Female (%)	10 (58.8)	17 (53.1)
Location		
Right colon (%)	12 (70.6)	8 (25.0)
Left colon (%)	5 (29.4)	24 (75.0)

### 2.3 *DKK1* inhibits cell proliferation and invasion in HCT116 cells

**FIGURE 3:**
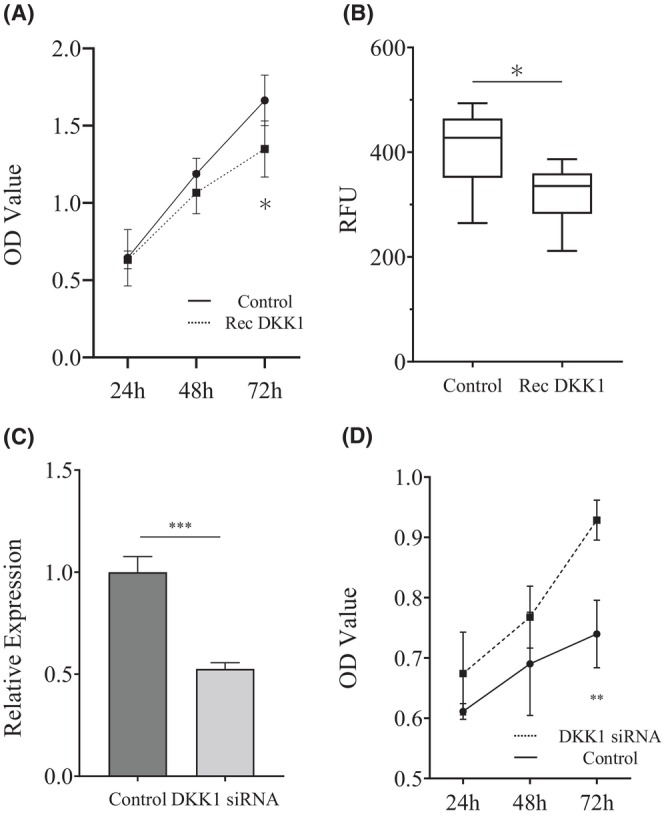
Functional evaluation of *DKK1* using human colon cancer cell line HCT116. (A) OD values obtained using the MTS assay after 24, 48, and 72 h of incubation of HCT116 cells with recombinant human *DKK1* protein. *n* = 6. (B) Cell invasion assay after incubation of HCT116 cells with recombinant human *DKK1* protein. *n* = 6. (C) Relative expression of *DKK1* after siRNA transfection using Lipofectamine 3000. (D) MTS assay in HCT116 cells at 24, 48, and 72 h after siRNA transfection. *n* = 4. *p < 0.05; **p < 0.01; ***p < 0.001. Rec *DKK1* = recombinant human *DKK1* protein.

Next, we examined the functional effects of *DKK1* on colorectal tumors using the human colon cancer cell line HCT116. In the MTS assay performed on HCT116 cells cultured with recombinant human *DKK1* protein, the mean optical density (OD) value of the protein‐added group was significantly lower (1.34, range 1.18–1.63) than that in the control group (1.66, 1.52–1.83), at 72 h after the culture (*p* = 0.010; Figure 3A). Moreover, the cell invasion ability of the protein‐added group significantly reduced compared to that in the control group (*p* = 0.043; Figure 3B)—the average fluorescent intensities of the two groups were 321 (range 211–387) and 411 (285–454) RFU, respectively. Next, we performed siRNA transfection to silence *DKK1* in HCT116 cells, which revealed significant suppression of *DKK1* expression in siRNA‐transfected cells compared to that in the control cells (*p* < 0.001; Figure 3C). Furthermore, the MTS assay after transfection revealed significantly increased average OD values in siRNA‐transfected cells (0.93, 0.89–0.97) compared to that in control cells (0.74, 0.67–0.81) at 72 h post‐transfection (*p* = 0.001; Figure 3D).

### 2.4 *DKK1* expression in culture supernatant during co‐culture and serum in patients with colorectal tumors

**FIGURE 4:**
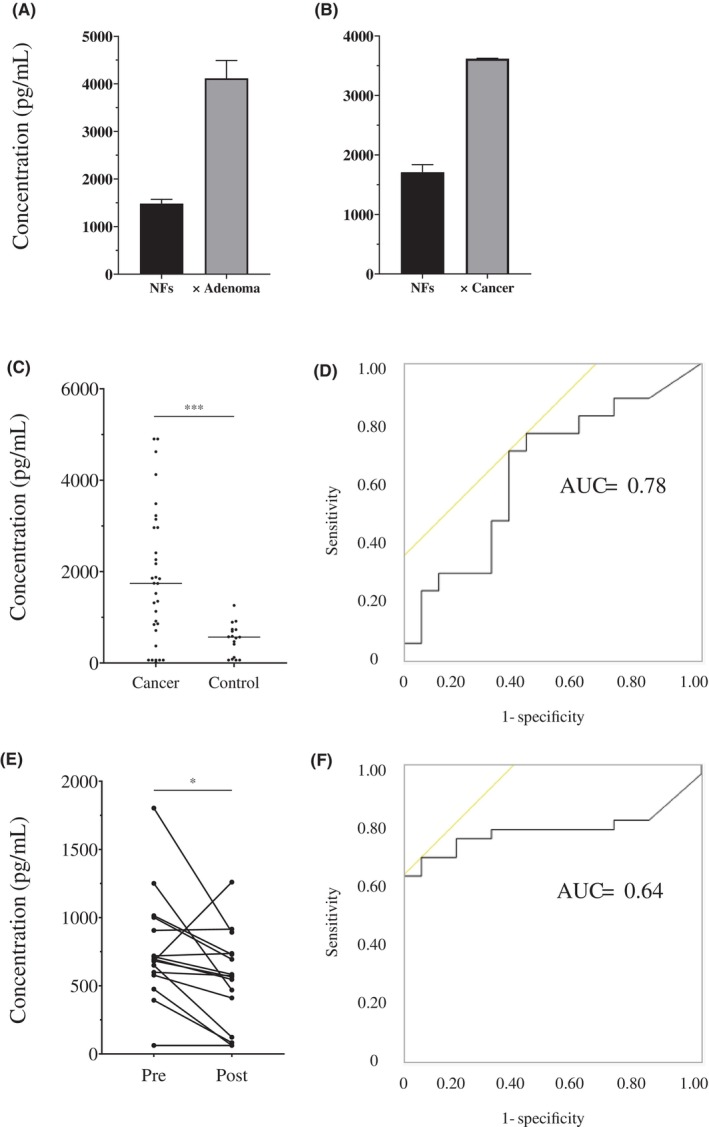
Serum DKK1 expression analysis. (A, B) ELISA of supernatant during co‐culture at Days 5–7; *n* = 2. Left: NFs monoculture, right: NFs co‐cultured with organoid (A) Using adenoma organoid. (B) Using cancer organoid. (C–F) Serum DKK1 expression in patients with colorectal tumors. (C) Comparison of serum DKK1 in samples obtained from patients with advanced cancer (*n* = 32) and control (*n* = 17). (D) ROC curve for (C), AUC = 0.78. (E) Comparison of serum DKK1 in paired serum samples from patients with advanced adenoma before and after treatment (*n* = 17). (F) ROC curve for (E), AUC = 0.64. In (C) and (D), *p < 0.05; ***p < 0.001. AUC, area under the curve; *DKK1*, Dickkopf‐1; post, post‐treatment; pre, pre‐treatment; ROC, receiver operating characteristic.

Next, we evaluated whether *DKK1* was elevated in the co‐culture medium. The concentration of *DKK1* was found to be increased by co‐culture with tumor organoids than when NFs were cultured alone (Figure 4A,B). Furthermore, as *DKK1* is expressed in colorectal tumor tissues since early tumor developmental stages, we measured serum DKK1 in patients with colorectal tumors to evaluate its usefulness as a biomarker. As shown in Figure 4C, the analysis revealed significantly higher serum DKK1 levels in the advanced cancer patient group (1863.5 pg/mL; 62.5–4902 pg/mL; *n* = 32) than in the control group (504.8 pg/mL; 62.5–1260 pg/mL; *n* = 17); the AUC was 0.78 (Figure 4D). Moreover, serum DKK1 concentration before and after treatment in patients with advanced adenoma (*n* = 17) showed a decrease in the after‐treatment group compared with that in the before‐treatment group (*p* = 0.023; Figure 4E); the AUC was 0.64 (Figure 4F).

## 3 MATERIALS AND METHODS

### 3.1 Research participants and organoid establishment

Colorectal tumors were defined as adenomas categories 3–4 and cancer category 5 according to the Vienna Classification.^23^, ^24^ Adenoma organoids were established by collecting tumor tissues from resection specimens at the time of endoscopic submucosal dissection (ESD) of category 3–4 tumors at Tohoku University Hospital (TUH), and normal epithelium organoids were established by collecting tissue from normal mucosa. Cancer organoids were established by collecting tumor tissues from resection specimens at the time of surgery of category 5 tumors at TUH.

Organoid cultures were established based on previously published protocols.^25^, ^26^ For tumor organoids, we used 100 ng/mL Recombinant Human Insulin‐Like Growth Factor‐I (BioLegend, San Diego, CA, USA) and 50 ng/mL Recombinant Human Fibroblast Growth Factor‐Basic (PeproTech, Cranbury, NJ, USA) instead of SB202190. For normal epithelium organoids, we used IntestiCult Organoid Growth Medium (STEMCELL Technologies, Vancouver, Canada) as the culture medium. The culture medium was replaced every 2–3 days, and organoids were passaged every week. For preparing the specimens for observation, ECM was removed using Cell Recovery Solution (Corning, Corning, NY, USA), coagulated in iPGell (NIPPON Genetics, Tokyo, Japan), fixed overnight in 4% paraformaldehyde (Wako, Osaka, Japan), embedded in paraffin blocks, sectioned using a microtome, and stained with Hematoxylin & Eosin (H&E). Images of the specimens were captured using the SLIDEVIEW VS200 Slide Scanner (Olympus Scientific Solutions, Tokyo, Japan) and observed.

### 3.2 Establishment of colonic NFs

Colonic NFs were established based on previously published protocols.^20^, ^27^, ^28^ To obtain the tissue, during en bloc resection of specimens after colorectal ESD, normal mucosa was excised and washed in PBS with 1% antibiotic‐antimycotic (Thermo Fisher Scientific, Waltham, MA, USA). Dulbecco's Modified Eagle Medium (DMEM)/Ham's F‐12 with L‐Glutamine and Phenol Red (Thermo Fisher Scientific) plus 1% Antibiotic‐Antimycotic and 20% fetal bovine serum (FBS; Cytiva, Tokyo, Japan) was used as the medium. As the demarcation between the tumor and non‐tumor regions of colorectal tumors is visibly discernible through the naked eye or endoscopic observation, we collected normal mucosa specifically from areas identified as tumor‐free by visual assessment. NFs were subjected to fluorescent immunostaining using Image‐iT Fixation/Permeabilization Kit (Thermo Fisher Scientific) and observed using a confocal laser microscope (C2si; Nikon Corporation, Tokyo, Japan). Anti‐α‐smooth muscle (*αSMA*; 14‐9760‐80; dilution 1:1000; Thermo Fisher Scientific) and anti‐E‐cadherin (610181; dilution 1:1000; Becton, Dickinson and Company, Franklin Lakes, NJ, USA) were used as primary antibodies, and Donkey anti‐Mouse IgG (H + L) Highly Cross‐Adsorbed Secondary Antibody, Alexa Fluor™ 488 (A‐21202, dilution 1:2000; Thermo Fisher Scientific) was used as a secondary antibody.

### 3.3 Co‐culture of organoids and NFs

To analyze the interaction between organoids and NFs, we employed a non‐contact co‐culture method. NFs were pre‐seeded at the bottom of 24‐well plate (Corning). Upon reaching confluence, organoids were suspended in Matrigel, and 10 μL of this suspension was placed in a cell culture insert (0.4 μm; Corning) containing the medium used for the establishment of each type of organoid (please see Section 2.1). The medium was changed every 3–4 days, and three NFs were used in each experiment. On the 10th day of culture, the diameter of organoids was measured: eight organoids were measured in order of size per field of view for a total of 64 organoids. To evaluate the significance of the changes in organoids during co‐culture, adenoma organoids were cultured in an NFs‐conditioned medium. Once the NFs seeded in the dish reached confluence, the medium was replaced with Advanced DMEM/F12 (Thermo Fisher Scientific) containing 1% penicillin–streptomycin (Thermo Fisher Scientific) and collected after 3 days to prepare the complete medium. The medium was replaced every 2–3 days, and the changes were observed under a microscope (CKX41; Olympus Scientific Solutions) on Day 7 of culture.

### 3.4 Assessment of cell viability

After co‐culture, the cell viability of organoids was evaluated using CellTiter‐Glo 3D Cell Viability Assay (Promega, Madison, WI, USA) and Luminoskan Ascent (Thermo Fisher Scientific) following the product instructions. Data were analyzed by measuring four wells each for co‐cultured and non‐co‐cultured organoids.

### 3.5 Microarray analysis of NFs

Co‐culture was performed, and RNA of NFs was extracted using ISOSPIN Cell & Tissue RNA (Nippon Gene, Tokyo, Japan) and suspended in RNase‐free water. Microarray analysis was performed using SurePrint G3 Human GE 8x60K Ver. 3.0 (Agilent Technologies, Santa Clara, CA, USA) following the product instructions. Data were quantified using the Feature Extraction software (Agilent Technologies) and normalized using the quantile method with R statistical software (R Foundation for Statistical Computing, Vienna, Austria).

### 3.6 Immunohistochemical staining of tissue specimens

Immunohistochemistry (IHC) was performed in endoscopically resected specimens obtained from TUH. Briefly, paraffin‐embedded sections were deparaffinized and activated using the microwave method with citrate buffer. Hydrogen peroxide was used to remove endogenous peroxidase activity. Primary antibodies, including Dickkopf‐1 (*DKK1*; ab109416; dilution 1:500; Abcam, Cambridge, UK), *DKK2* (ab38594; dilution 1:200; Abcam), and secreted frizzled related protein‐1 (*SFRP1*; ab126613; dilution 1:100; Abcam), were used. Dako EnVision+ System‐ HRP Labeled Polymer Anti‐Rabbit (Agilent Technologies) was used as the secondary antibody, and DAB substrate (Takara Bio, Shiga, Japan) was used for detection. The specimen images were captured using the SLIDEVIEW VS200 slide scanner (Olympus Scientific Solutions). Three gastroenterology specialists observed the images of the entire preparation and scored staining in the tumor area. For scoring, staining intensity and percent of immunoreactivity were recorded following an established protocol,^29^ and the two scores were multiplied to obtain a *DKK1* expression score (0–9 points). Tumor areas of immunostained specimens were evaluated separately in the tumor adenoducts and stroma. Specimens were classified as normal mucosa, nonadvanced adenoma, advanced adenoma,^30^ or advanced cancer.

### 3.7 Culture of human colon cancer cell line

The human colon cancer cell line HCT116 was obtained from the American Type Culture Collection. The cells were cultured in high‐glucose DMEM supplemented with L‐Glutamine, Phenol Red (Wako), 10% FBS (Cytiva), and 1% penicillin–streptomycin (Thermo Fisher Scientific). Cells were passaged at a density of 1 × 10^5^ cells/mL and cultured at 37°C under a 5% carbon dioxide vapor phase.

### 3.8 Investigation of changes in cell proliferation and invasiveness by addition of *DKK1* recombinant protein

To analyze the function of *DKK1* in colon cancer cell lines, HCT116 cells at a density of 4.0 × 10^3^ cells per well were seeded in 96‐well plates and cultured in the medium supplemented with 200 ng/mL recombinant human *DKK1* protein (R&D Systems, Minneapolis, MN, USA). Using CellTiter 96 AQ_ueous_ One Solution Cell Proliferation Assay (Promega), the MTS assay was performed simultaneously on the six wells following the product instructions, using Multiskan FC Microplate Reader (Thermo Fisher Scientific). Next, we used CytoSelect Cell Invasion Assay Kit (Cell Biolabs, San Diego, CA, USA) following the product instructions. The medium inside and outside the insert was similarly adjusted to contain 200 ng/mL of recombinant protein. Absorbance measurements were performed using SpectraMax M2e (Molecular Devices, San Jose, CA, USA). Experiments were conducted concurrently in six wells for both the recombinant protein‐added group and the control group without the recombinant protein.

### 3.9 SiRNA transfection to silence *DKK1* in HCT116 cells and real‐time PCR

Small interfering RNA (siRNA) transfection was performed on HCT116 cells using Lipofectamine 3000 Reagent (Thermo Fisher Scientific) to silence *DKK1* (Thermo Fisher Scientific, siRNA ID: HSS117947). After transfection, the RNA of HCT116 cells was extracted using ISOSPIN Cell & Tissue RNA (Nippon Gene). The extracted RNA was used to synthesize cDNA using SuperScript IV VILO Master Mix with ezDNase Enzyme (Thermo Fisher Scientific), and real‐time PCR was performed using TaqMan Universal PCR Master Mix (Thermo Fisher Scientific) and Light Cycler 96 system (Roche Diagnostics) in triplicates to confirm transfection efficiency. *DKK1* Hs00183740_m1 assay (Thermo Fisher Scientific) was used to measure the expression of *DKK1*. The amplified products were analyzed using the 2^−ΔΔCt^ method. Furthermore, the MTS assay was performed using HCT116 cells simultaneously on four wells after siRNA transfection.

### 3.10 ELISA

The expression of *DKK1* in the culture supernatant during co‐culture was analyzed. Furthermore, paired serum before and after advanced adenoma treatment and serum from patients with advanced cancer were used to measure serum DKK1 levels. Post‐treatment of advanced adenoma was defined as no evidence of tumor recurrence and no residual neoplastic lesions under lower gastrointestinal endoscopy 6 months after colorectal ESD for advanced adenoma (control group). DuoSet ELISA Ancillary Reagent Kit 2 (R&D Systems) and Human DKK‐1 DuoSet ELISA Kit (R&D Systems) were used for the experiments, and absorbance was measured using SpectraMax M2e (Molecular Devices). Experiments were performed in duplicate.

### 3.11 Statistical analysis

Statistical analyses were performed using JMP Pro 16.1.0 (SAS Institute Inc, Cary, NC, USA).

Statistically significant differences were determined using *t*‐test, Welch's *t*‐test, *χ* ^2^ test, and paired *t*‐test. Furthermore, the receiver operating characteristic (ROC) curve and area under the curve (AUC) were used to evaluate the diagnostic performance of *DKK1* in each experiment. In each analysis, *p* < 0.05 was considered statistically significant.

## 4 DISCUSSION

In this study, we used a co‐culture system of NFs and colon organoids to examine their effects on the proliferative potential of organoids. The findings of this study suggest the possibility that the response of NFs differs between adenoma and cancer. Several studies have reported the co‐culture of colon cancer cell lines with CAFs or NFs, suggesting the proliferative potential of the cell lines.^31^, ^32^, ^33^, ^34^ However, experiments using organoids are limited. Hirokawa et al. reported that normal epithelium organoids showed enhanced proliferative potential when co‐cultured with NFs,^20^ which was consistent with our study. To the best of our knowledge, we could not find any reports using adenoma organoids, which showed an apparent change in their appearance and a remarkable increase in their diameter in our study. As the NFs and organoids were cultivated following a non‐contact co‐culture method, these changes were speculated to be due to liquid factor‐mediated intercellular signaling from NFs. However, culturing adenoma organoids with NF conditioned medium did not lead to morphological changes. These findings suggest that co‐culture led to a bidirectional cross‐talk and the release of liquid factors rather than liquid factors being constantly released as they are by NFs. The existence of such cross‐talk between epithelial cells and fibroblasts in vivo has been previously described.^35^, ^36^ Kalluri and Zeisberg^35^ showed that fibroblasts adjacent to the basement membrane become activated when tumor cells disrupt the basement membrane and initiate invasion, which has further been speculated to be dependent on integrin and soluble factor‐mediated cross‐talk.^36^ Taken together, the results of this study could be attributed to such bidirectional action; however, further studies are essential to precisely understand the underlying mechanisms. Several genetic abnormalities occur in the adenoma‐carcinoma sequence, and the study demonstrates the potential of adenoma organoids to mimic TME and aid in the identification of the peripheral factors for tumor malignancy. Additionally, NFs, which are also present around precancerous lesions, respond differently to adenomas and carcinomas, suggesting the diverse roles of fibroblasts in tumorigenesis.

Gene expression analysis of NFs after co‐culture showed that *DKK1* was progressively upregulated in co‐culture with adenoma and cancer organoids. *DKK1* is a secreted protein identified and a well‐known antagonist of Wnt signaling and an inhibitor of the β‐catenin pathway.^37^, ^38^ The Wnt/β‐catenin pathway is one of the major pathways in colorectal cancer, and aberrant activation of this pathway is associated with cell proliferation, invasion, and cell resistance, making it a potential therapeutic target.^39^ Several studies have reported the association of *DKK1* with colon cancer. Studies related to organoids with three‐dimensional structures have also been conducted, with recent studies reporting on the activation of CAFs with tumor organoids secreting *DKK1*.^40^ However, only a few reports describing *DKK1* expression in fibroblasts themselves have been found for non‐neoplastic diseases.^41^ In the present study, *DKK1* suppressed the proliferative potential of colon cancer cell lines, which was restored by silencing of *DKK1*.

In this study, immunostaining for *DKK1* using clinical specimens showed that staining of the tumor stroma gradually decreased with progressive malignancy, while staining of tumor adenoducts increased. While a previous report indicated a decrease in *DKK1* expression during the transition from normal epithelium to colorectal cancer,^29^ conflicting with our study, it is more plausible that *DKK1* expression in serum increased with tumor development, consistent with our findings, suggesting stronger histological expression. Immunostaining of tumors at different stages of development revealed *DKK1* expression in the tumor stroma starting from the early stages of tumor development, and it may be involved in early responses of fibroblasts to tumors.

Nevertheless, some reports indicate that serum DKK1 levels are elevated in patients with advanced colorectal cancer,^42^, ^43^, ^44^ whereas others report no elevation.^45^, ^46^ In the present study, elevated serum DKK1 levels were observed in patients with advanced cancer compared to those with endoscopically confirmed clean colon. Furthermore, analysis of serum before and after treatment in patients with advanced adenomas showed a significant decrease in serum DKK1 levels after treatment. These findings suggest that *DKK1* is secreted into the blood at the early stages of the tumor.

The present study has a few limitations. We considered using *DKK1* as a candidate gene for gene transfer to organoids and NFs; however, it was difficult due to technical problems and the slow growth rate of NFs. Therefore, we used cancer cell lines instead. In addition, immunostaining and ELISA were based on the short‐term collection at a single institution, and further accumulation of such data is desirable. As described above, most previous reports were related to advanced cancers, but in this study, new findings were obtained by conducting experiments on endoscopically curable early‐stage tumors, such as adenomas.

In conclusion, during the development of colorectal tumors, fibroblasts express *DKK1* from the early stage of tumorigenesis and may function in a tumor‐suppressive manner. These results also suggest that the effects of fibroblasts may differ depending on the degree of tumor progression. Although Wnt signaling, including *DKK1* and the peritumoral microenvironment, has been studied for many years in colorectal cancer, there are still some unexplored areas, and further studies are needed to elucidate the pathogenesis.

## AUTHOR CONTRIBUTIONS

**Yushi Inomata:** Conceptualization (equal); data curation (equal); investigation (equal); methodology (equal); project administration (equal); writing – original draft (equal); writing – review and editing (equal). **Masatake Kuroha:** Conceptualization (equal); data curation (equal); formal analysis (supporting); funding acquisition (supporting); investigation (equal); methodology (equal); project administration (equal); resources (supporting); software (supporting); supervision (equal); validation (equal); visualization (equal); writing – original draft (equal); writing – review and editing (equal). **Yusuke Shimoyama:** Methodology (equal); supervision (equal). **Takeo Naito:** Data curation (equal); supervision (equal); validation (equal). **Rintaro Moroi:** Supervision (equal); validation (equal); visualization (equal). **Hisashi Shiga:** Investigation (equal); methodology (equal); project administration (equal). **Yoichi Kakuta:** Conceptualization (equal); formal analysis (equal); funding acquisition (equal); investigation (equal); methodology (equal); supervision (equal); validation (equal). **Hideaki Karasawa:** Methodology (equal); resources (equal); validation (equal). **Shinobu Onuma:** Resources (equal); supervision (equal). **Yoshitaka Kinouchi:** Supervision (equal); writing – original draft (supporting); writing – review and editing (supporting). **Atsushi Masamune:** Methodology (equal); supervision (equal); validation (equal); writing – original draft (equal); writing – review and editing (equal).

## FUNDING INFORMATION

The authors did not receive support from any organization for the submitted work.

## CONFLICT OF INTEREST STATEMENT

The authors declare no competing interests. All authors have read the journal's policy on conflicts of interest and the authorship agreement.

## ETHICS STATEMENT

This study was conducted as per the Declaration of Helsinki and approved by the Ethics Committee of Tohoku University Graduate School of Medicine (nos. 2022–1‐119, 2022–1‐169, 2022–1‐249).

## PATIENT CONSENT STATEMENT

All patients provided written informed consent before participation.

## Supporting information


Data S1.

## Data Availability

The data that support the findings of this study are available from the corresponding author, Masatake Kuroha, upon reasonable request.
